# Stomach and Intestines

**Published:** 1905-11-11

**Authors:** 


### STOMACH AND INTESTINES.

**Clinical Methods.**—The determination of the functional condition of the stomach by the examination of its contents is an aid to diagnosis which is perhaps not sufficiently practised in this country, partly, no doubt, owing to the unwillingness of patients to allow the passage of a stomach tube. That valuable information may be obtained by this method is, however, undeniable. A simple procedure has been described by Willcox,[1] who points out that it is not always necessary to pass a tube, as the vomit of patients suffering from gastric disturbances answers equally well. Willcox, as a test meal, recommends a pint of weak tea, not more than one ounce of milk, and sugar as desired, or a pint of thin arrowroot or other cereal gruel, made with water, two ounces of milk, and a little sugar if desired. To either of these should be added a round of thin buttered toast. This test meal appears to have many advantages. It is not unpalatable, being, indeed, very like an ordinary early breakfast, and is free from various substances which he enumerates as likely to interfere with the chemical examination of the gastric contents. These should be withdrawn in an hour and a half, and not diluted. A careful microscopical examination should then be made of the solid portion, and its general appearance and reaction noted. A special chemical examination for total acidity and total active HCl. is then undertaken. Willcox points out that it is not sufficient to determine the presence of chemically free HCl. The point to be ascertained is the actual secretory power of the stomach with regard to HCl., so that the amount of free acid and also that which has already combined with organic matter in the food must be estimated together. Inorganic combinations of HCl. need not be considered, as they are introduced into the stomach with food and not formed within it. For the estimation of total acidity Willcox recommends titration with decinormal barium hydrate, using phenolphthalein as an indicator. The filtered gastric contents must be first freed from $CO_2$, by passage through them of a current of pure air. The estimation of what is termed "physiologically active" HCl. is by a modification of Volhard's method, the accuracy of which the author has determined on numerous occasions. The procedure, which is simple and convenient, consists in first estimating the total chlorides, combined organic and inorganic, and then driving off the free HCl. and splitting up the organic salts, leaving only the residue of inorganic chlorides, which are estimated in the same way. The difference, of course, shows the amount of "physiologically active" HCl. present. Willcox then reviews other methods—namely, those of Sjogvist, Leo, Toepfer, Shufflebotham, Ham and McLeod, Dawson and Sidney Martin. Most of these, he finds, are inaccurate in some respect, except the last named, which, barring a few details, coincides with his own. Qualitative tests for HCl. are then dealt with, the preference being given to Guntzberg's. He points out, however, the importance of using freshly-prepared reagents, and that the presence of a considerable amount of proteid matter, by combining with the free acid, will interfere with the test. The dimethylazoamidobenzene reaction and the tests with tropeolin OO and Congo red he has found unsatisfactory. This careful paper should certainly do much to encourage the examination of the stomach contents in all doubtful cases; it is interesting to note that Willcox has collected sixty cases in which such an examination has been made at St. Mary's Hospital, and his conclusions are that in gastric and duodenal ulcer the total acidity is high (0.2 per cent. to 0.3 per cent.), mostly due to "active" HCl.; organic acids being generally absent, and peptone only present, if at all, in small amount. In temporary dyspepsia the total acidity is about normal (0.1 per cent. to 0.2 per cent.), mostly "active" HCl.; organic acids may be present in small amount, and albumose or peptone often in considerable quantities. In carcinoma Willcox finds that much depends on the position of the growth. If it involves the cardiac end, the total acidity is low (under 0.04 per cent.), free HCl. is never present, and "active" HCl. only in small amount (0.02 per cent.), or completely absent. Organic acid is generally present with mucin, but little or no albumose or peptone. In growth of the pyloric portion the total acidity is generally higher (0.05 per cent. to 0.1 per cent.), and "active" HCl. may reach 0.1 per cent. Mucin, albumin, and quite large amounts of peptone are often found.

Willcox's results certainly show that the examination is clinically worth the time expended upon it, though it is probable that negative results are not so reliable as positive ones; in certain anæmias for example HCl. may be absent or present only in very small quantities. Whether the laboratory examination of the fæces is capable of yielding results proportionate to expenditure of time and trouble must be still considered doubtful. If they are to be examined the method advocated by Stern[2] will be found useful; but apart from the natural disinclination which many practitioners would feel to undertaking an exhaustive examination, it may be doubted whether the knowledge to be gained is a sufficiently important aid in diagnosis or treatment. Stern himself admits that individual differences are so great in a complex process like intestinal digestion that no "normal" or "standard" fæces can be logically described. His method consists in placing the individual to be tested on a special diet for a couple of days, and examining the solid excreta passed thirty hours after the beginning of the test period. He divides his examination into three parts, the physico-chemical, the macroscopical, and the microscopical. The first includes observation of smell, colour, reaction and consistence, and examination for albumin, peptone (absent in health), hydrobilirubin (normally present) and fermentation (frothy motions smelling of butyric acid). The last two are devoted to determination of mucus, concretions, and the debris of food. Three microscopical preparations are made, one untreated, one treated with acetic acid (for estimation of fats), and one with Lugol's solution (for estimation of unaltered starch, yeast cells, and other vegetable organisms).

[1] Lancet, June 10, 1905, p. 1566. [2] Med. Rec., July 8, 1905, p. 45.

*(To be continued.)*

